# The identification of novel targets of miR-16 and characterization of their biological functions in cancer cells

**DOI:** 10.1186/1476-4598-12-92

**Published:** 2013-08-14

**Authors:** Xin Yan, Hongwei Liang, Ting Deng, Kegan Zhu, Suyang Zhang, Nan Wang, Xueyuan Jiang, Xueliang Wang, Rui Liu, Ke Zen, Chen-Yu Zhang, Yi Ba, Xi Chen

**Affiliations:** 1 Tianjin Medical University Cancer Institute and Hospital, Huanhuxi Road, Tiyuanbei, Tianjin 300060, China; 2 Jiangsu Engineering Research Center for microRNA Biology and Biotechnology, State Key Laboratory of Pharmaceutical Biotechnology, School of Life Sciences, Nanjing University, 22 Hankou Road, Nanjing 210093, China

**Keywords:** microRNA, miR-16, MAP7, PRDM4, CDS2

## Abstract

**Background:**

In eukaryotes, miR-16 is an important microRNA (miRNA) that is involved in numerous biological processes. However, it is not fully understood how miR-16 executes its physiological functions. In the present study, we aimed to identify novel miR-16 targets and study their biological functions.

**Methods:**

Candidate target genes of miR-16 were screened by microarray analysis of mRNA levels in several cancer cell lines with enhanced miR-16. Three bioinformatics algorithms, including TargetScan, PicTar, and miRanda, were used in combination to calculate the miR-16 targets. The expression levels of miR-16 and target mRNA were examined by relative quantification RT-PCR, and the expression levels of target protein were detected by Western blot. Luciferase reporter plasmids were constructed to confirm direct targeting. The effect of miR-16 and target gene on cell viability was evaluated using MTT assays. The effects of miR-16 and target gene on apoptosis and cell cycle distribution were evaluated by flow cytometry analysis.

**Results:**

By overexpressing miR-16 in several cancer cell lines and measuring global mRNA levels using microarray analysis, we identified 27 genes that may be regulated by miR-16. After the bioinformatics filtering process, 18 genes were selected as candidate miR-16 targets. Furthermore, we experimentally validated three of these candidates, MAP7 (microtubule-associated protein 7), PRDM4 (PR domain containing 4) and CDS2 (CDP-diacylglycerol synthase 2), as direct targets of miR-16. Finally, we demonstrated that miR-16 targeting MAP7 played a critical role in regulating proliferation but not apoptosis and cell cycle progression in cancer cells.

**Conclusion:**

In summary, the present study identifies several novel miR-16 targets and illustrates a novel function of miR-16 targeting MAP7 in modulating proliferation in cancer cells.

## Introduction

microRNAs (miRNAs) are a class of endogenous noncoding RNAs, typically 22 nucleotides in length, that function primarily by targeting the 3′-untranslated region (3′-UTR) of specific mRNAs and hence silence gene expression by either translational repression or direct mRNA degradation [1,2]. Through these post-transcriptional gene regulation mechanisms, miRNAs regulate a wide range of biological processes, including cell proliferation and differentiation, migration, apoptosis, development and metabolism [1,2].

The number of miRNAs encoded by the genomes of different organisms varies dramatically, and more than 2000 miRNAs have been identified in humans [1,2]. Some of these miRNAs have attracted special attention for their involvement in the initiation, progression and metastasis of human cancers [3-5]. One particularly well-studied example is the ubiquitously expressed and highly conserved miR-16, one of the first miRNAs to be linked to human malignancies [6]. Evidence indicates that miR-16 can modulate the cell cycle, inhibit cell proliferation, promote cell apoptosis and suppress tumorigenicity both *in vitro* and *in vivo*[7]. These effects can be explained by several targets of miR-16: the anti-apoptotic gene Bcl-2 (B-cell lymphoma 2) [8]; numerous genes involved in the G1-S transition, such as cyclin D1, cyclin D3, cyclin E1 and CDK6 (cyclin-dependent kinase 6) [9-11]; and genes involved in the Wnt signaling pathway, such as WNT3A (wingless-type MMTV integration site family, member 3A) [11]. Consistently, miR-16 is frequently deleted and/or downregulated in many types of cancer, such as chronic lymphocytic leukemia [6,12], prostate cancer [11] and lung cancer [13].

Given the importance of miR-16 during tumorigenesis, the aim of the present study is to identify new miR-16 targets and study their biological functions in cancer cells. By overexpressing miR-16 in several cancer cell lines and measuring global mRNA levels through microarray platforms, we identified a large number of transcripts that are potentially regulated by miR-16. We further confirmed MAP7 (microtubule-associated protein 7), PRDM4 (PR domain containing 4) and CDS2 (CDP-diacylglycerol synthase 2) as direct targets of miR-16. The mechanism through which miR-16 executes its functions in cancer cells was also investigated in this study.

## Methods

### Cells

The human lung adenocarcinoma cell line A549, human breast cancer cell line MCF-7, human epithelial carcinoma cell line HeLa, human colon adenocarcinoma cell line SW480 and human embryonic kidney cell line HEK-293 were purchased from the Shanghai Institute of Cell Biology, Chinese Academy of Sciences (Shanghai, China). These cells were maintained in RPMI 1640 medium (Gibco, CA, USA) or DMEM medium (Gibco) supplemented with 10% fetal bovine serum (FBS) (Gibco). Cells were grown at 37°C in a humidified atmosphere with 5% CO_2_.

### miR-16 overexpression or knockdown

miR-16 overexpression was achieved by transfecting cells with pre-miR-16 (a synthetic RNA oligonucleotide duplex mimicking miR-16 precursor), while miR-16 knockdown was achieved by transfecting cells with anti-miR-16 (a chemically modified single-stranded antisense oligonucleotide designed to specifically target against mature miR-16). Scrambled negative control RNA (pre-miR-control and anti-miR-control) served as negative control. Synthetic RNA molecules, including pre-miR-16, anti-miR-16 and scrambled negative control RNA, were purchased from GenePharma (Shanghai, China). Cells were seeded on 60-mm dishes and were transfected the following day using Lipofectamine 2000 (Invitrogen, Carlsbad, CA, USA) according to the manufacturer’s instructions. For each well, equal doses (200 pmol) of pre-miR-control, pre-miR-16, anti-miR-control or anti-miR-16 were added. Cells were harvested 24 h after transfection.

### RNA isolation and relative quantification RT-PCR

Total RNA was extracted from the cultured cells using TRIzol Reagent (Invitrogen) according to the manufacturer’s instructions. Assays to quantify mature miR-16 were carried out using Taqman microRNA probes (Applied Biosystems, Foster City, CA, USA) according to the manufacturer’s instructions, with slight modification. Briefly, 1 μg of total RNA was reverse-transcribed to cDNA using AMV reverse transcriptase (TaKaRa, Dalian, China) and a stem-loop RT primer (Applied Biosystems). The reaction conditions were: 16°C for 30 min, 42°C for 30 min, 85°C for 5 min. Real-time PCR was performed using a TaqMan PCR kit on an Applied Biosystems 7300 Sequence Detection System (Applied Biosystems). The reactions were incubated in a 96-well optical plate at 95°C for 10 min, followed by 40 cycles of 95°C for 15 s and 60°C for 1 min. All reactions were run in triplicate. After the reactions, the threshold cycles (C_T_) values were determined using fixed threshold settings, and the mean C_T_ was determined from the triplicate PCRs. In the experiments presented here, a comparative C_T_ method was used to compare each condition with controls. miRNA expression in cells was normalized to that of the U6 snRNA. The amount of miR-16 relative to the internal control U6 was calculated with the equation 2^-△ △ CT^, in which △ △ C_T_ = (C_T miR - 16_ - C_T U6_)_target_ - (C_T miR - 16_ - C_T U6_)_control_.

For relative quantification RT-PCR analysis of MAP7, PRDM4 and β-actin mRNA, 1 μg of total RNA was reverse-transcribed to cDNA with oligo dT and Thermoscript (TaKaRa, Dalian, China) in the reaction conditions: 42°C for 60 min and 70°C for 10 min. Then real-time PCR was performed on an Applied Biosystems 7300 Sequence Detection System (Applied Biosystems) using SYBR green dye (Invitrogen). The 20-μl PCR reaction included 1 μl RT product, 1 × QuantiTect SYBR green PCR Master Mix, and 0.5 μM each sense and antisense primers. The reactions were incubated in a 96-well plate at 95°C for 5 min, followed by 40 cycles of 95°C for 30 s, 60°C for 30 s, and 72°C for 30 s. All reactions were run in triplicate. After the reactions, the C_T_ values were determined using fixed threshold settings. The relative amount of MAP7 and PRDM4 mRNA was normalized to β-actin mRNA. The sequences of the primers are as follows: MAP7 (sense): 5′-AAACTCTTTGTAACACCACCTGA-3′; MAP7 (antisense): 5′-GATGGAGATACAGCCCTTCG-3′; PRDM4 (sense): 5′-CGAAAGATTCATGGTGGAAA-3′; PRDM4 (antisense): 5′-TAAGGTGGTGGAGGTAGGGT-3′; β-actin (sense): 5′-AGGGAAATCGTGCGTGAC-3′; and β-actin (antisense): 5′-CGCTCATTGCCGATAGTG-3′.

### Microarray procedures

The commercially available 22 K Human Genome Array was purchased from the CapitalBio Corporation (Beijing, China). Labeling, hybridization, washing, and scanning were performed according to the standard operating procedure provided by CapitalBio. Briefly, total RNA was used to synthesize cDNA in an *in vitro* transcription reaction. cDNA was fluorescently labeled by Cy5 or Cy3-CPT using the Klenow enzyme. After hybridization, non-specifically bound molecules were removed from the microarray with two consecutive washes (0.2% SDS and 2 × SSC at 42°C for 5 minutes followed by 0.2% SSC for 5 minutes at room temperature). Subsequently, the arrays were scanned with a LuxScan 10KA confocal laser scanner (CapitalBio Corporation), and the obtained images were analyzed using LuxScan Version 3.0 (CapitalBio Corporation) employing the LOWESS normalization method.

### miR-16 target prediction

The miRNA target prediction and analysis was performed with the algorithms from TargetScan (http://www.targetscan.org/) PicTar (http://pictar.mdc-berlin.de/) and miRanda (http://www.microrna.org/).

### Western blotting

MAP7 and PRDM4 protein levels were quantified by western blot analysis of whole cell extracts using antibodies against MAP7 and PRDM4. These samples were normalized by blotting with an antibody against α-tubulin. Anti-MAP7 (NBP1-46240) antibody was purchased from Novus (CO, USA), and anti-PRDM4 (sc-15254) and anti-α-tubulin (B-7) antibodies were purchased from Santa Cruz Biotechnology (CA, USA).

### Luciferase assay

The entire 3′-UTRs of human CDS2, PRDM4, MAP7, PPP1R11, CHUK, LAMP2 and SLC35A4 were amplified from human genomic DNA using PCR. The PCR products were inserted into the p-MIR-report plasmid (Ambion). Efficient insertion was confirmed by sequencing. For luciferase reporters containing mutant CDS2, PRDM4 and MAP7 3′-UTRs, the sequences that interact with bases 2–8 of the miR-16 seed sequence were mutated. For luciferase reporter assays, cells were cultured in 6-well plates, and each well was transfected with 2 μg of firefly luciferase reporter plasmid, 2 μg of β-galactosidase expression plasmid (Ambion), and equal amounts of scrambled negative control RNA, pre-miR-16, or anti-miR-16 using Lipofectamine 2000 (Invitrogen). The β-galactosidase plasmid was used as a transfection control. At 24 h post-transfection, cells were assayed using luciferase assay kits (Promega, Madison, WI, USA). The data depicted are representative of three independent experiments performed on different days.

### Plasmid construction and siRNA interference assay

A mammalian expression plasmid encoding the human MAP7 open reading frame (pReceiver-M02-MAP7) was purchased from GeneCopoeia (Germantown, MD, USA). An empty plasmid served as a negative control. The siRNA (sequence: CAGAUUAGAUGUCACCAAUTT) targeting human MAP7 cDNA was designed and synthesized by Invitrogen (Carlsbad, CA, USA). A scrambled siRNA (Stealth™ RNAi negative control kit, Invitrogen, Carlsbad, CA, USA) that could not target human MAP7 cDNA was included as a negative control. Plasmid and siRNA were transfected into A549 cells using Lipofectamine 2000 (Invitrogen) according to the manufacturer’s instructions. Total RNA and protein was isolated at 24 h post-transfection. The MAP7 mRNA and protein expression levels were assessed by relative quantification RT-PCR and western blotting.

### Cell viability assay

A549 cells were plated at 2.5 × 10^3^ cells per well in 96-well plates and incubated overnight in DMEM medium supplemented with 10% FBS. After transfection, 20 μl 3-(4,5-dimethylthiazol-2-yl)-2,5-diphenyl tetrazolium bromide (MTT) (5 mg/mL) was added into a corresponding test well and incubated for 4 h. The supernatant was then discarded, and 200 μL of DMSO was added to each well to dissolve the precipitate. Optical density (OD) was measured at a wavelength of 570 nm.

### Apoptosis assays

Apoptosis was detected using an Annexin V-FITC/propidium iodide (PI) staining assay. A549 cells were cultured in 12-well plates and transfected with 40 pmol of pre-miR-16 or siRNA of MAP7 to induce apoptosis. Pre-miR-control and control siRNA served as negative controls. Cells were cultured overnight with both serum-containing complete medium and serum-depleted medium; the attached cells and floating cells were then harvested. Flow cytometry analysis of apoptotic cells was carried out using an Annexin V-FITC/PI staining kit (BD Biosciences, CA, USA). After washes with cold PBS, the cells were resuspended in binding buffer (100 mM HEPES, pH 7.4, 100 mM NaCl, and 25 mM CaCl_2_) followed by staining with Annexin V-FITC/PI at room temperature in darkness for 15 min. Apoptotic cells were then evaluated by gating PI and Annexin V-positive cells on a fluorescence-activated cell-sorting (FACS) flow cytometer (BD Biosciences, San Jose, CA). All experiments were performed in triplicate.

### Cell cycle assay

Cells were harvested, washed once with PBS, and fixed in 70% ethanol overnight. Staining for DNA content was performed with 50 mg/ml propidium iodide and 1 mg/ml RNase A for 30 min. Analysis was performed on a FACS flow cytometer (BD Biosciences) with Cell Quest Pro software. Cell cycle modeling was performed with Modfit 3.0 software (Verity Software House, Topsham, ME).

### Statistical analysis

All presented images of western blotting and cell cycle assay are representative of at least three independent experiments. Relative quantification RT-PCR, luciferase reporter, and cell viability assays were performed in triplicate, and each experiment was repeated three to five times. The data shown are the mean ± SD of at least three independent experiments. Statistical significance was considered at p < 0.05 using the Student’s t-test.

## Results

### The identification of candidate miR-16 targets by microarray analysis and bioinformatics algorithms

**Figure 1:**
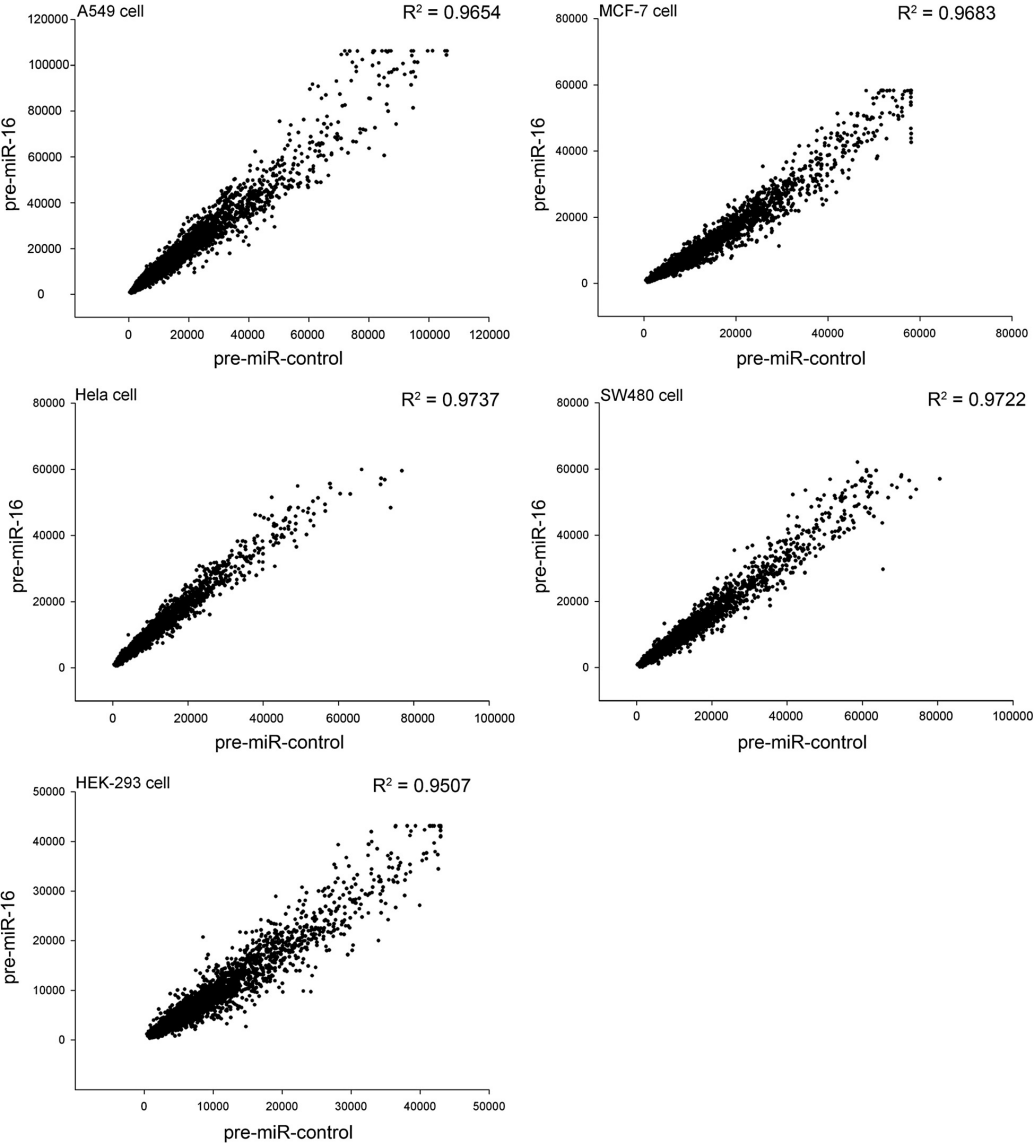
**Scatter plots showing mRNA expression in pre-miR-16-transfected cells versus control cells.** A549, MCF-7, HeLa, SW480 and HEK-293 cells were seeded on 60-mm dishes and transfected the following day with Lipofectamine 2000. For each well, 200 pmol of pre-miR-16 or pre-miR-control were added. At 24 h post-transfection, mRNA from these cells was subjected to microarray analysis.

Animal miRNAs were originally believed to block translational processes without affecting transcript levels [1-3]. However, recent evidence has changed this view, showing that target mRNA degradation is a widespread effect of miRNA-based regulation that alone accounts for most of the repression mediated by miRNAs in mammalian cell cultures [14]. Inspired by this mode of miRNA action, we postulated that the high-throughput mRNA microarray assays capable of detecting such effects at the mRNA level would provide a promising avenue for miRNA target identification. Considering that miR-16 is a ubiquitously expressed miRNA frequently downregulated in many types of cancer, we chose several cell lines with very different origins for microarray analysis to identify the common targets of miR-16. The selected human cell lines included A549 (lung adenocarcinoma cell), MCF-7 (breast cancer cell), HeLa (epithelial carcinoma cell), SW480 (colon adenocarcinoma cell) and HEK-293 (embryonic kidney cell). We transfected A549, MCF-7, HeLa, SW480 and HEK-293 cells with equal doses of pre-miR-16 (miRNA mimic) or pre-miR-control (scrambled miRNA mimic, as a negative control) and then surveyed mRNA transcripts that were inversely expressed relative to miR-16 using microarray analysis. The results showed that although most mRNAs were not influenced by transfection with miR-16, some mRNAs were downregulated in pre-miR-16-transfected cells compared with control cells (Figure 1). To reduce the false positive rate and obtain a more accurate assessment of the genuine miR-16 targets, only the mRNAs that were downregulated by a factor of at least 1.5 (0.66-fold downregulation) in at least four cell lines were considered as putative miR-16 targets. Finally, 27 mRNAs were selected for further analysis (Table 1).

**Table 1:** mRNAs identified as downregulated in cells overexpressing miR-16

**Gene name**	**A549**	**MCF-7**	**Hela**	**SW480**	**HEK-293**	**Mean fold**
ARL2	0.4438	0.3506		0.5400	0.1817	0.3790
CDS2	0.5127	0.3022	0.5133		0.3045	0.4082
ANAPC13		0.3843	0.6239	0.3393	0.4607	0.4520
NUPL1		0.3315	0.6230	0.5340	0.3525	0.4602
MAP7	0.6168	0.4428	0.6220	0.5151	0.2027	0.4799
ARG2	0.4384	0.5793		0.4731	0.4845	0.4938
RTN4	0.6063	0.4024		0.5277	0.4415	0.4945
RARS	0.6351	0.4953	0.5922		0.2686	0.4978
PRDM4	0.6458	0.3892	0.5288	0.3809	0.5978	0.5085
SPRYD3	0.6232	0.3945	0.6387	0.5158	0.3778	0.5100
ATF6	0.5601	0.5654		0.5141	0.4159	0.5139
TMEM109		0.5647	0.5863	0.4650	0.4396	0.5139
RPS6KA3	0.5621	0.4471	0.6515	0.5522	0.3611	0.5148
BCR		0.6217	0.5980	0.4826	0.3719	0.5185
KIF3B	0.6569	0.5500	0.5970	0.3850	0.4665	0.5311
ENTPD6	0.5493	0.5145	0.5929	0.4446	0.5748	0.5352
CCND3		0.4777	0.5767	0.4867	0.6042	0.5363
ARHGDIA		0.5038	0.6295	0.5048	0.5103	0.5371
GABARAPL1		0.5492	0.6459	0.6228	0.3693	0.5468
PLEKHB2		0.4670	0.6040	0.5178	0.6149	0.5509
FLJ11149		0.4721	0.6577	0.6297	0.5081	0.5669
DNAJC5		0.5794	0.6497	0.4857	0.5730	0.5720
ZNF622		0.6543	0.6059	0.6394	0.4159	0.5789
ANXA11	0.6548	0.5811		0.4592	0.6410	0.5840
VPS33B	0.6586	0.6122		0.5394	0.5359	0.5865
ANLN		0.4597	0.6433	0.6247	0.6247	0.5881
PTH2	0.5264	0.6352		0.5819	0.6600	0.6009

Next, three algorithms, including TargetScan [15], PicTar [16], and miRanda [17], were used in combination to calculate whether the downregulated mRNAs were candidate miR-16 targets. Only the mRNAs predicted as miR-16 targets by at least two of the above-mentioned algorithms were considered positive. In total, 18 mRNAs were identified as candidate miR-16 targets (Table 2).

**Table 2:** Candidate miR-16 targets predicted by TargetScan, PicTar and miRanda

**Gene name**	**TargetScan**	**PicTar**	**miRanda**	**Result**
ARL2	4	5	1	Positive
CDS2	2	2	1	Positive
ANAPC13	1	0	0	
NUPL1	0	0	0	
MAP7	2	2	2	Positive
ARG2	0	0	0	
RTN4	1	0	1	Positive
RARS	0	0	0	
PRDM4	1	1	1	Positive
SPRYD3	4	0	2	Positive
ATF6	1	0	1	Positive
TMEM109	2	0	2	Positive
RPS6KA3	2	0	1	Positive
BCR	1	1	1	Positive
KIF3B	1	0	1	Positive
ENTPD6	2	0	0	
CCND3	2	0	1	Positive
ARHGDIA	4	5	1	Positive
GABARAPL1	3	0	2	Positive
PLEKHB2	2	0	0	
FLJ11149	0	0	0	
DNAJC5	2	0	0	
ZNF622	1	1	1	Positive
ANXA11	1	0	1	Positive
VPS33B	2	0	1	Positive
ANLN	1	0	1	Positive
PTH2	0	0	0	

Numbers in the table indicate the putative binding sites of miR-16 in the 3′-UTR of target genes.

### Identification of direct miR-16 targets by luciferase reporter screening

**Figure 2:**
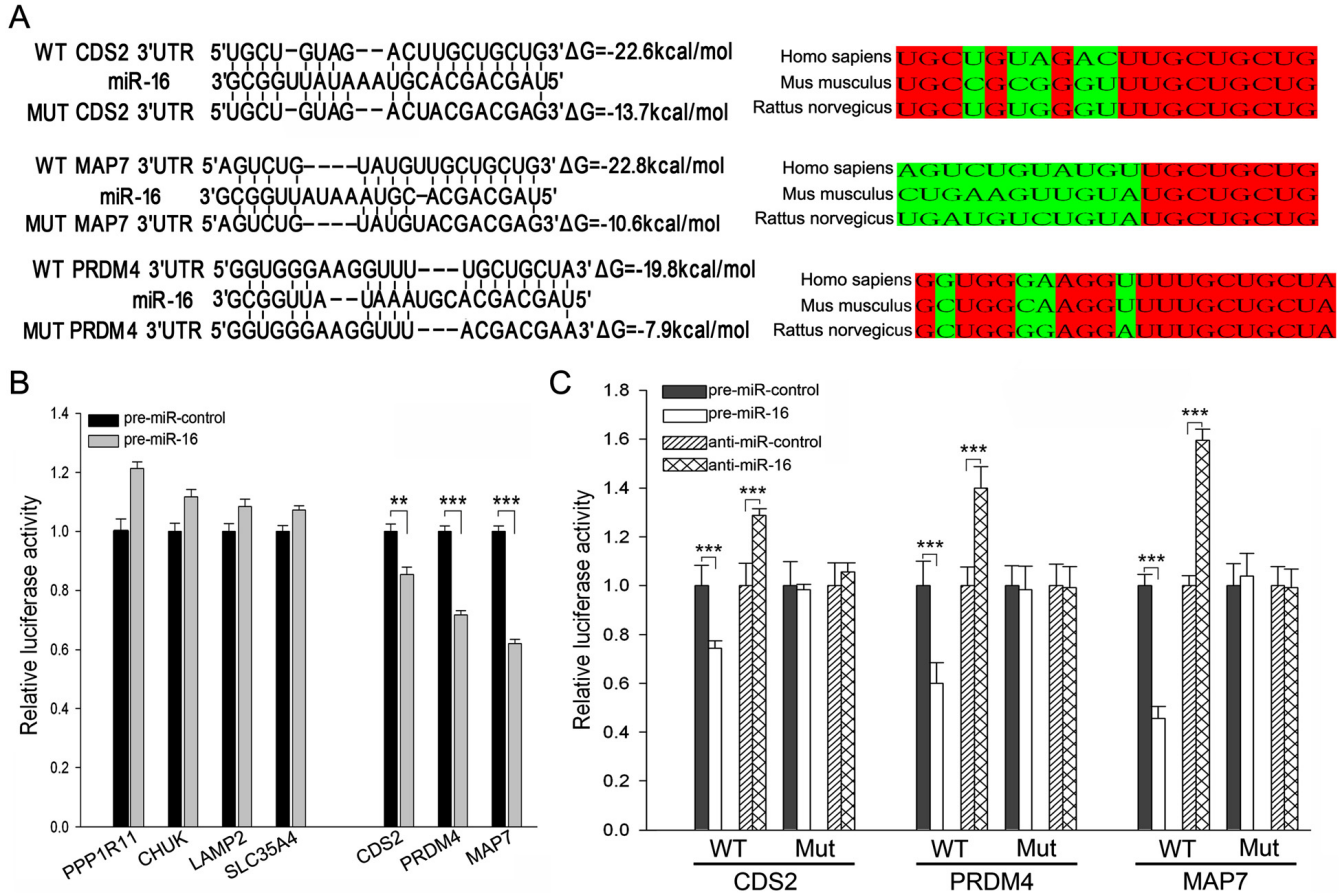
**Identification of direct miR-16 targets by luciferase reporter screening. (A)** Schematic description of the hypothetical duplexes formed by interactions between miR-16 and the wild-type (WT) or mutant (MUT) 3′-UTRs of CDS2, PRDM4 and MAP7. For WT 3′-UTRs, perfect base pairing between the “seeds” (the core sequence that encompasses the first 2–8 bases of the mature miRNA) and cognate targets was noted. For MUT 3′-UTRs, the sequence that interacts with the 2–8 bases of miR-16 were mutated. The predicted minimum free energy values of each hybrid are indicated, and the miR-16 binding sequences at the 3′-UTR of CDS2, PRDM4 and MAP7 are highly conserved across species. **(B)** The identification of direct miR-16 targets by luciferase reporter screening. Firefly luciferase reporters containing the CDS2, PRDM4 or MAP7 3′-UTRs were co-transfected with pre-miR-16 or pre-miR-control into A549 cells. Luciferase reporters containing the 3′-UTRs of PPP1R11, CHUK, LAMP2 and SLC35A4 served as negative controls. At 24 h post-transfection, cells were assayed using luciferase assay kits. The results are presented as the mean ± SD of three independent experiments (** p < 0.01; *** p < 0.001). **(C)** Direct recognition of the 3′-UTRs of CDS2, PRDM4 and MAP7 by miR-16. Firefly luciferase reporters containing either WT or MUT CDS2, PRDM4 or MAP7 3′-UTRs were co-transfected with pre-miR-16, pre-miR-control, anti-miR-16 or anti-miR-control into A549 cells. At 24 h post-transfection, cells were assayed using luciferase assay kits. The results presented are the mean ± SD of three independent experiments (*** p < 0.001).

However, transcript-based expression analysis and computational predictions alone cannot directly measure the actual miRNA-target interaction. Therefore, a luciferase reporter assay was conducted to screen for target genes that were directly controlled by miR-16 through the 3′-UTR. Three genes, including MAP7, PRDM4 and CDS2, were selected. The predicted interaction between miR-16 and these genes is illustrated in Figure 2A. The entire 3′-UTRs of these genes were fused into a downstream position of the firefly luciferase gene in a reporter plasmid. Luciferase reporters containing the 3′-UTRs of PPP1R11 (protein phosphatase 1 regulatory subunit 11), CHUK (conserved helix-loop-helix ubiquitous kinase), LAMP2 (lysosomal-associated membrane protein 2) and SLC35A4 (solute carrier family 35, member A4) were generated and served as negative controls (PPP1R11, LAMP2 and SLC35A4 contain putative miR-16 binding sites but were not significantly altered by transfection with miR-16, while CHUK does not have a candidate miR-16 binding sequence). The resulting plasmids were transfected into A549 cells combined with pre-miR-16 or pre-miR-control. The luciferase activities were assayed 36 h after transfection. As shown in Figure 2B, overexpression of miR-16 significantly decreased the luciferase activities of the reporter containing the 3′-UTRs of MAP7, PRDM4 and CDS2, whereas the negative control reporters were unaffected by miR-16 in A549 cells.

Furthermore, we introduced point mutations into the corresponding seed complementary sites in the 3′-UTR of MAP7, PRDM4 and CDS2 to eliminate the predicted binding by miR-16 (Figure 2A). Luciferase reporters containing either wild-type or mutant MAP7, PRDM4 and CDS2 3′-UTRs were co-transfected with pre-miR-16, pre-miR-control, anti-miR-16 (miRNA antisense) or anti-miR-control (scrambled miRNA antisense as negative control) into A549 cells. While the overexpression of miR-16 decreased the luciferase activity, the inhibition of miR-16 resulted in a significant increase in the luciferase activity, and mutations in the seed complementary sites almost fully rescued the repression of reporter activity by miR-16 (Figure 2C). These results demonstrate that miR-16 can directly recognize the 3′-UTRs of MAP7, PRDM4 and CDS2 and mediate the post-transcriptional inhibition of these genes.

### The downregulation of MAP7 and PRDM4 expression by miR-16

**Figure 3:**
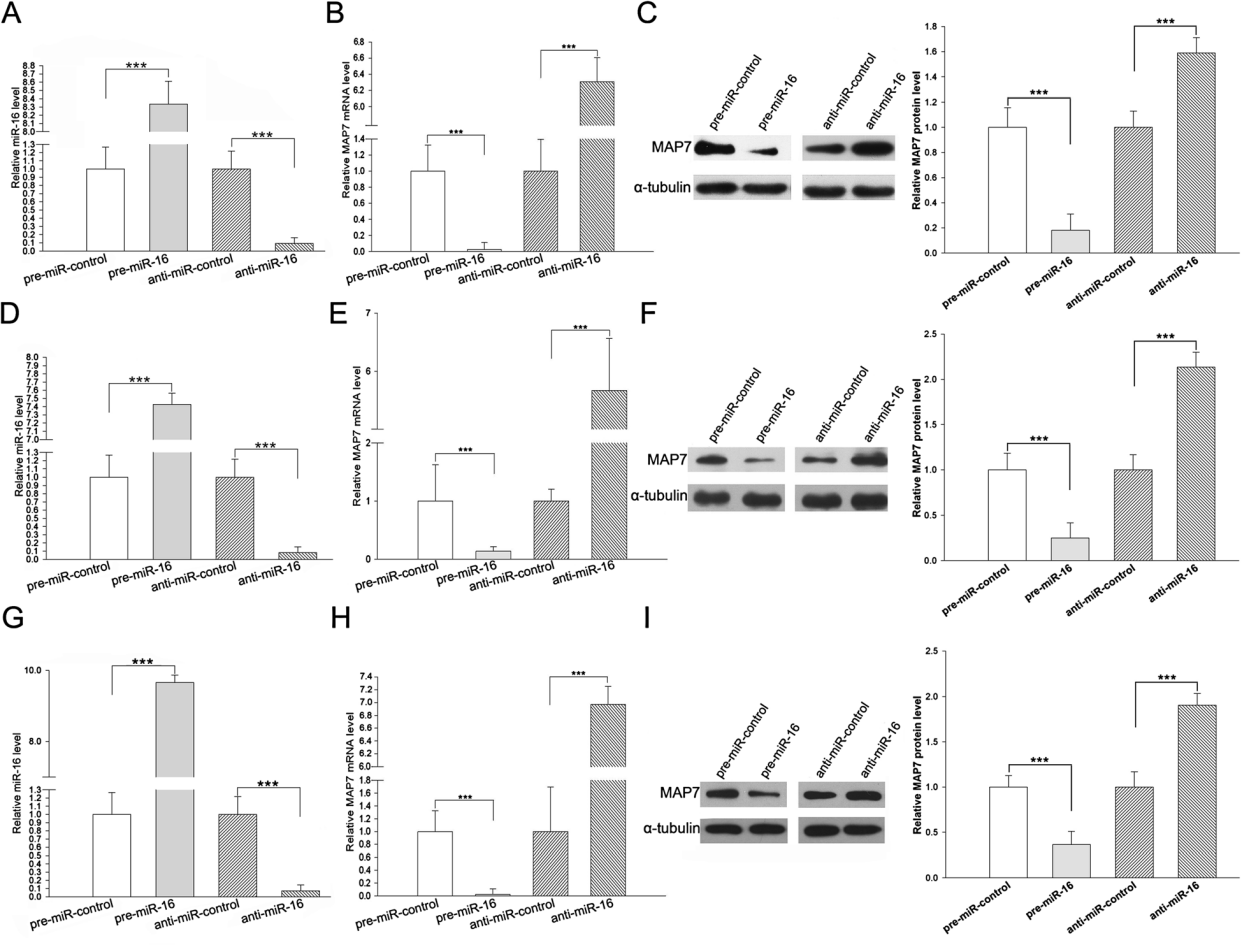
**Regulation of MAP7 expression by miR-16 at both the transcript and protein levels. (A, D and G)** Relative quantification RT-PCR analysis of miR-16 levels in A549 (A), Hela (D) and MCF-7 **(G)** cells treated with pre-miR-16, pre-miR-control, anti-miR-16 or anti-miR-control. The results presented are the mean ± SD of three independent experiments (*** p < 0.001). **(B, E and H)** Relative quantification RT-PCR analysis of MAP7 mRNA levels in A549 **(B)**, Hela **(E)** and MCF-7 **(H)** cells treated with pre-miR-16, pre-miR-control, anti-miR-16 or anti-miR-control. The results presented are the mean ± SD of three independent experiments (*** p < 0.001). **(C, F and I)** Western blot analysis of MAP7 protein levels in A549 **(C)**, Hela **(F)** and MCF-7 **(I)** cells treated with pre-miR-16, pre-miR-control, anti-miR-16 or anti-miR-control. Left panel: representative image; right panel: quantitative analysis (*** p < 0.001).

We then determined whether the overexpression or knockdown of miR-16 had an impact on MAP7 and PRDM4 expression. We transfected A549, HeLa and MCF-7 cells with equal doses of pre-miR-16, pre-miR-control, anti-miR-16 or anti-miR-control, and analyzed the expression levels of MAP7 mRNA by relative quantification RT-PCR at 24 h post-transfection. The expression of miR-16 was abolished by the introduction of anti-miR-16, whereas pre-miR-16 significantly increased miR-16 levels in A549, HeLa and MCF-7 cells (Figure 3, A, D and G). Cells transfected with pre-miR-16 showed reduced levels of MAP7 mRNA relative to cells transfected with pre-miR-control; in contrast, the expression levels of MAP7 mRNA were significantly increased in anti-miR-16-transfected cells compared with those transfected with anti-miR-control (Figure 3, B, E and H). Furthermore, we repeated the above experiments and determined the expression of MAP7 protein by western blotting at 24 h post-transfection. The expression levels of the MAP7 protein were significantly abolished by the introduction of pre-miR-16, whereas cells transfected with pre-miR-control maintained a considerable amount of MAP7 protein; in contrast, anti-miR-16 significantly increased the expression levels of MAP7 protein in A549, HeLa and MCF-7 cells (Figure 3, C, F and I). Moreover, we found that the PRDM4 mRNA and protein levels were inversely correlated to miR-16 in A549 cells (Additional file 1: Figure S1, A and B). These results demonstrate that miR-16 regulates the expression of MAP7 and PRDM4 at both the transcript and protein levels.

### The role of miR-16 in regulating MAP7 during tumorigenesis

**Figure 4:**
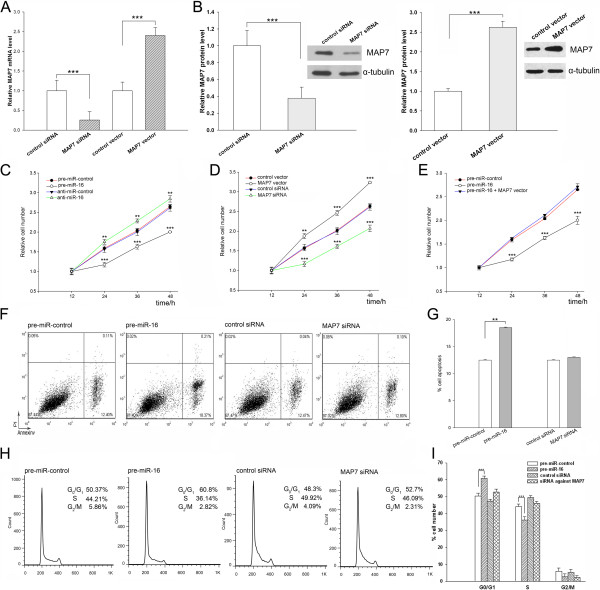
**The role of miR-16 targeting MAP7 in the regulation of proliferation, apoptosis and cell cycle progression. (A and B)** Efficient overexpression or knockdown of MAP7 expression. For knockdown of MAP7, siRNA against MAP7 and a scrambled control siRNA were transfected into A549 cells. For overexpression of MAP7, MAP7 overexpressing plasmid and an empty plasmid were transfected into A549 cells. Cells were harvested at 24 h post-transfection. MAP7 mRNA and protein levels were assessed by relative quantification RT-PCR **(A)** and Western blotting **(B)**. Left panel: quantitative analysis; right panel: representative image. **(C)** MTT cell viability assay at 12, 24, 36, and 48 h after transfection of A549 cells with equal doses of pre-miR-control, pre-miR-16, anti-miR-control or anti-miR-16. **(D)** MTT cell viability assay at 12, 24, 36, and 48 h after transfection of A549 cells with equal doses of control siRNA or siRNA against MAP7 or equal doses of empty plasmid or MAP7 overexpressing plasmid. **(E)** MTT cell viability assay at 12, 24, 36, and 48 h after transfection of A549 cells with equal doses of pre-miR-control, pre-miR-16, or pre-miR-16 along with the MAP7 overexpressing plasmid. **(F)** A549 cells transfected with equal doses of pre-miR-control, pre-miR-16, control siRNA or siRNA against MAP7 were labeled with FITC-Annexin V/PI, and serum deprivation-induced apoptosis was measured by flow cytometry. **(G)** Quantification of the apoptotic cells in panel F. **(H)** A549 cells were transfected with equal doses of scrambled ncRNA or pre-miR-16 or equal doses of control siRNA or siRNA against MAP7. Cell cycle profiles were analyzed using flow cytometry. Shown in the panel are histograms of cell numbers (y axis) against DNA content (x axis) determined by measuring fluorescence intensity. **(I)** Quantification of the percentages of cells in the G0/G1, S, and G2/M phases in panel **(H)** (mean ± SD; ** p < 0.01; *** p < 0.001).

We next focused on studying the role of miR-16 in regulating MAP7 and PRDM4. Because miR-16 is known to be involved in regulation of proliferation, apoptosis and cell cycle progression, we investigated whether the overexpression or knockdown of miR-16, MAP7 or PRDM4 would have an impact on these cellular phenotypes in A549 cells. To knock down MAP7 and PRDM4, siRNAs against MAP7 or PRDM4 were transfected into A549 cells. To overexpress MAP7 and PRDM4, recombinant plasmids designed to specially express the full-length open reading frame of MAP7 and PRDM4 without the miR-16–responsive 3′-UTRs were constructed and transfected into A549 cells. The efficient overexpression or knockdown of MAP7 (Figure 4, A and B) and PRDM4 (Additional file 1: Figure S1, C and D) is shown.

In supporting the notion that miR-16 is essential in repressing proliferation, A549 cells transfected with anti-miR-16 showed stimulation of cell proliferation; in contrast, overexpression of miR-16 had an opposite effect on cell proliferation (Figure 4C). Furthermore, we assessed the role of MAP7 and PRDM4 on cell proliferation. A549 cells transfected with a MAP7 overexpressing plasmid proliferated at a significantly higher rate, whereas the knockdown of MAP7 by siRNA significantly reduced proliferation (Figure 4D). Finally, compared to cells transfected with pre-miR-16, cells transfected with pre-miR-16 and MAP7 overexpressing plasmid exhibited significantly higher proliferation rates (Figure 4E), suggesting that miR-16-resistant MAP7 could rescue the suppression of MAP7 by miR-16. These results demonstrate that miR-16 can inhibit cell proliferation by silencing MAP7. On the other hand, no differences were observed in proliferation rates between the cells transfected with control siRNA or siRNA against PRDM4 (Additional file 1: Figure S1E). The results suggest that miR-16 could modulate cell proliferation by downregulating genes other than PRDM4.

Next, we investigated apoptosis in cells with enhanced miR-16 or silenced MAP7 or PRDM4 by flow cytometry analysis. Statistically significantly more apoptotic cells were observed in the pre-miR-16-transfected cells compared with pre-miR-control-transfected cells (Figure 4, F and G). However, no differences in the levels of apoptosis were observed in cells transfected with negative control siRNA or siRNA against MAP7 (Figure 4, F and G). Likewise, silencing of PRDM4 had no effect on apoptosis in A549 cells (Additional file 1: Figure S1, F and G). These results suggest that miR-16 could trigger apoptosis by downregulating genes other than MAP7 and PRDM4.

Finally, we investigated cell cycle distribution in cells with enhanced miR-16 or silenced MAP7 or PRDM4 by flow cytometry analysis. Compared with cells transfected with pre-miR-control, cells transfected with pre-miR-16 triggered an accumulation of cells in the G0/G1 stage, whereas the numbers of cells in the S and G2/M phases decreased (Figure 4, H and I). Transfection with siRNA against MAP7 partially yielded the phenotype generated by overexpression of miR-16, but the effects were not significant, featured by a lower G0/G1 cell accumulation but a higher S and G2/M cell accumulation (Figure 4, H and I). Moreover, silencing of PRDM4 had no effect on cell cycle progression of A549 cells (Additional file 1: Figure S1, H and I). The results suggest that miR-16 might negatively regulate cell cycle progression from the G0/G1 phase to the S phase by silencing genes other than MAP7 and PRDM4.

## Discussion

Although the number of known miRNAs is continuously increasing, information regarding their precise cellular function remains limited. One of the major challenges in understanding the functions of a specific miRNA is to identify its genuine target genes. To date, only a few miRNAs have been assigned target mRNAs. In the present study, we aimed to identify the genuine targets of miR-16, a miRNA that has long been thought to be implicated in tumorigenesis. By using microarray analysis to globally screen the expression patterns of transcripts in several cell lines with overexpressed miR-16 and by combining bioinformatics programs to select genuine miR-16 targets from the differentially regulated genes, we identified a panel of 18 candidate miR-16 target genes. Actually, the same strategy has been used in our earlier study [18]. In that study, in order to identify the common targets of miR-16, we performed the microarray analysis in A549, MCF-7 and HEK-293 cells with either enhanced or silenced miR-16 [18]. In this study, we re-performed the microarray analysis in A549, MCF-7 and HEK-293 cells (we have re-performed the whole procedure of the microarray analysis, from preparation of new mRNA samples to analysis of new data), as well as in additional HeLa and SW480 cells with enhanced miR-16. There are some overlap of microarray data between this study and our earlier study. Among the 27 mRNAs identified as downregulated in cells overexpressing miR-16, nine (ARL2, CDS2, ANAPC13, MAP7, ARG2, RTN4, RARS, SPRYD3 and RPS6KA3) were also selected by our earlier study. The consistency between these two studies further demonstrates the robustness of our strategy to identify the common miR-16 targets. On the other hand, the additional 18 mRNAs identified as downregulated in this study were not observed in the prior analysis. This may be due to the different criteria we used to select miR-16 targets. In our previous study, only the mRNAs that showed consistent downregulation (fold change < 0.66) in all three cell lines were considered as candidate miR-16 targets. In the present study, the mRNAs that showed downregulation (fold change < 0.66) in at least four cell lines were considered as putative miR-16 targets. Thus, for mRNAs such as NUPL1, TMEM109 and CCND3, because they did not show significant downregulation in A549 cells, they were not included as candidate miR-16 targets in our previous study. However, in the present study they were included because they showed downregulation in other four cell lines. We have provided a table to summarize the comparison findings between these two studies (Additional file 1: Table S1).

Subsequently, we randomly selected three genes, MAP7, PRDM4 and CDS2, from the candidate 18 genes and used a luciferase reporter screen to experimentally demonstrate that all three of these genes are directly targeted by miR-16. Indeed, we demonstrated that miR-16 regulates the expression of MAP7 and PRDM4 at both the transcript and protein levels through several biological approaches. The results demonstrate that our strategy of combining global gene expression analysis with bioinformatics prediction may provide good selection criteria to aid in miRNA target identification.

We also showed that miR-16 could regulate cell proliferation but not apoptosis and cell cycle progression in A549 cells by silencing MAP7. However, how MAP7 regulates cell proliferation is currently unknown. MAP7 is a microtubule-associated protein that is predominantly expressed in cells of epithelial origin [19]. Microtubule-associated proteins are involved in microtubule dynamics, which are essential for many important cellular processes including cell division, motility and differentiation [19]. MAP7 has been shown to modulate microtubule functions [19]. Thus, MAP7 may function as a proliferation promoter through its role in the microtubule dynamics. Furthermore, the role of MAP7 in cancer progression is also unclear. In support of a potential role for MAP7 in metastatic growth, this gene was recently identified as one out of only fifteen that was highly upregulated in metastatic endometrial cancer using a 22 K Affymetrix array [20]. Moreover, high MAP7 expression has been associated with tumor recurrence and poor prognosis in Stage II colon cancer patients [21]. Here, we showed that MAP7 had the potential to accelerate proliferation, but not influence apoptosis and cell cycle progression in A549 cells. Although we did not investigate the consequence of miR-16 targeting MAP7 in other cancer cell lines, as miR-16 could regulate the expression of MAP7 in A549, HeLa and MCF-7 cells, we speculate that miR-16 might have similar cellular functions through silencing MAP7. Therefore, the downregulation of miR-16 in cancer cells would, in theory, relieve the suppression of miR-16 on MAP7, which in turn accelerates tumorigenesis. The modulation of MAP7 protein level by miR-16 may explain, at least in part, why the downregulation of miR-16 can promote cancer progression. Additional studies are necessary to fully elucidate the exact roles of miR-16 in regulating MAP7 in other cancer cells. Additional studies are necessary to uncover the clinical effects of miR-16 in regulating MAP7 during cancer progression.

In this study, we uncovered multiple targets of miR-16 by combining mRNA microarray profiles with bioinformatics analysis. We further experimentally validated MAP7 as a direct target of miR-16 and demonstrated that this targeting plays a critical role in regulating proliferation in cancer cells.

## Competing interests

The authors declare that they have no competing interests.

## Authors’ contributions

XC, YB and CYZ designed research and analyzed data. XY, HL, TD, KZ, SZ, NW and XJ performed experiments. XW and RL performed statistical analysis. KZ made contributions to the conception and design of experiments. XC and YB wrote the manuscript. All authors read and approved the final manuscript.

## Supplementary Material

Additional file 1: Figure S1 Regulation of PRDM4 expression by miR-16. (A) Relative quantification RT-PCR analysis of PRDM4 mRNA levels in A549 cells treated with pre-miR-16, pre-miR-control, anti-miR-16 or anti-miR-control. (B) Western blot analysis of PRDM4 protein levels in A549 cells treated with pre-miR-16, pre-miR-control, anti-miR-16 or anti-miR-control. Left panel: representative image; right panel: quantitative analysis. (C and D) Efficient overexpression or knockdown of PRDM4 expression. For knockdown of PRDM4, siRNA against PRDM4 and a scrambled control siRNA were transfected into A549 cells. For overexpression of PRDM4, PRDM4 overexpressing plasmid and an empty plasmid were transfected into A549 cells. Cells were harvested at 24 h post-transfection. PRDM4 mRNA and protein levels were assessed by relative quantification RT-PCR (C) and Western blotting (D). Left panel: quantitative analysis; right panel: representative image. (E) MTT cell viability assay at 12, 24, 36, and 48 h after transfection of A549 cells with equal doses of control siRNA or siRNA against PRDM4. (F) A549 cells transfected with equal doses of pre-miR-control, pre-miR-16, control siRNA or siRNA against PRDM4 were labeled with FITC-Annexin V/PI, and serum deprivation-induced apoptosis was measured by flow cytometry. (G) Quantification of the apoptotic cells in panel F. (H) A549 cells were transfected with equal doses of control siRNA or siRNA against PRDM4. Cell cycle profiles were analyzed using flow cytometry. Shown in the panel are histograms of cell numbers (y axis) against DNA content (x axis) determined by measuring fluorescence intensity. (I) Quantification of the percentages of cells in the G0/G1, S, and G2/M phases in panel H. (mean ± SD; ** p < 0.01; *** p < 0.001). Table S1. mRNAs identified as downregulated in cells overexpressing miR-16 by this study and our previous study. Click here for file
