# Supplementary material for: The identification of novel targets of miR-16 and characterization of their biological functions in cancer cells
Source: Mol Cancer. 2013 Aug 14;12:92. doi: 10.1186/1476-4598-12-92 (PMC3751425; doi:10.1186/1476-4598-12-92)
Supplement: Additional file 1: Figure S1 — Regulation of PRDM4 expression by miR-16. (A) Relative quantification RT-PCR analysis of PRDM4 mRNA levels in A549 cells treated with pre-miR-16, pre-miR-control, anti-miR-16 or anti-miR-control. (B) Western blot analysis of PRDM4 protein levels in A549 cells treated with pre-miR-16, pre-miR-control, anti-miR-16 or anti-miR-control. Left panel: representative image; right panel: quantitative analysis. (C and D) Efficient overexpression or knockdown of PRDM4 expression. For knockdown of PRDM4, siRNA against PRDM4 and a scrambled control siRNA were transfected into A549 cells. For overexpression of PRDM4, PRDM4 overexpressing plasmid and an empty plasmid were transfected into A549 cells. Cells were harvested at 24 h post-transfection. PRDM4 mRNA and protein levels were assessed by relative quantification RT-PCR (C) and Western blotting (D). Left panel: quantitative analysis; right panel: representative image. (E) MTT cell viability assay at 12, 24, 36, and 48 h after transfection of A549 cells with equal doses of control siRNA or siRNA against PRDM4. (F) A549 cells transfected with equal doses of pre-miR-control, pre-miR-16, control siRNA or siRNA against PRDM4 were labeled with FITC-Annexin V/PI, and serum deprivation-induced apoptosis was measured by flow cytometry. (G) Quantification of the apoptotic cells in panel F. (H) A549 cells were transfected with equal doses of control siRNA or siRNA against PRDM4. Cell cycle profiles were analyzed using flow cytometry. Shown in the panel are histograms of cell numbers (y axis) against DNA content (x axis) determined by measuring fluorescence intensity. (I) Quantification of the percentages of cells in the G0/G1, S, and G2/M phases in panel H. (mean ± SD; ** p < 0.01; *** p < 0.001). Table S1. mRNAs identified as downregulated in cells overexpressing miR-16 by this study and our previous study. [file 1476-4598-12-92-S1.pdf]

The identification of novel targets of miR-16 and characterization of their biological functions in cancer cells

Additional file 1

Figure S1. Regulation of PRDM4 expression by miR-16. (A) Relative quantification RT-PCR analysis of PRDM4 mRNA levels in A549 cells treated with pre-miR-16, pre-miR-control, anti-miR-16 or anti-miR-control. (B) Western blot analysis of PRDM4 protein levels in A549 cells treated with pre-miR-16, pre-miR-control, anti-miR-16 or anti-miR-control. Left panel: representative image; right panel: quantitative analysis. (C and D) Efficient overexpression or knockdown of PRDM4 expression. For knockdown of PRDM4, siRNA against PRDM4 and a scrambled control siRNA were transfected into A549 cells. For overexpression of PRDM4, PRDM4 overexpressing plasmid and an empty plasmid were transfected into A549 cells. Cells were harvested at 24 h post-transfection. PRDM4 mRNA and protein levels were assessed by relative quantification RT-PCR (C) and Western blotting (D). Left panel: quantitative analysis; right panel: representative image. (E) MTT cell viability assay at 12, 24, 36, and 48 h after transfection of A549 cells with equal doses of control siRNA or siRNA against PRDM4. (F) A549 cells transfected with equal doses of pre-miR-control, pre-miR-16, control siRNA or siRNA against PRDM4 were labeled with FITC-Annexin V/PI, and serum deprivation-induced apoptosis was measured by flow cytometry. (G) Quantification of the apoptotic cells in panel F. (H) A549 cells were transfected with equal doses of control siRNA or siRNA against PRDM4. Cell cycle profiles were analyzed using flow cytometry. Shown in the panel are histograms of cell numbers (y axis) against DNA content (x axis) determined by measuring fluorescence intensity. (I) Quantification of the percentages of cells in the G0/G1, S, and G2/M phases in panel H. (mean ± SD; ** $p < 0.01$; *** $p < 0.001$).

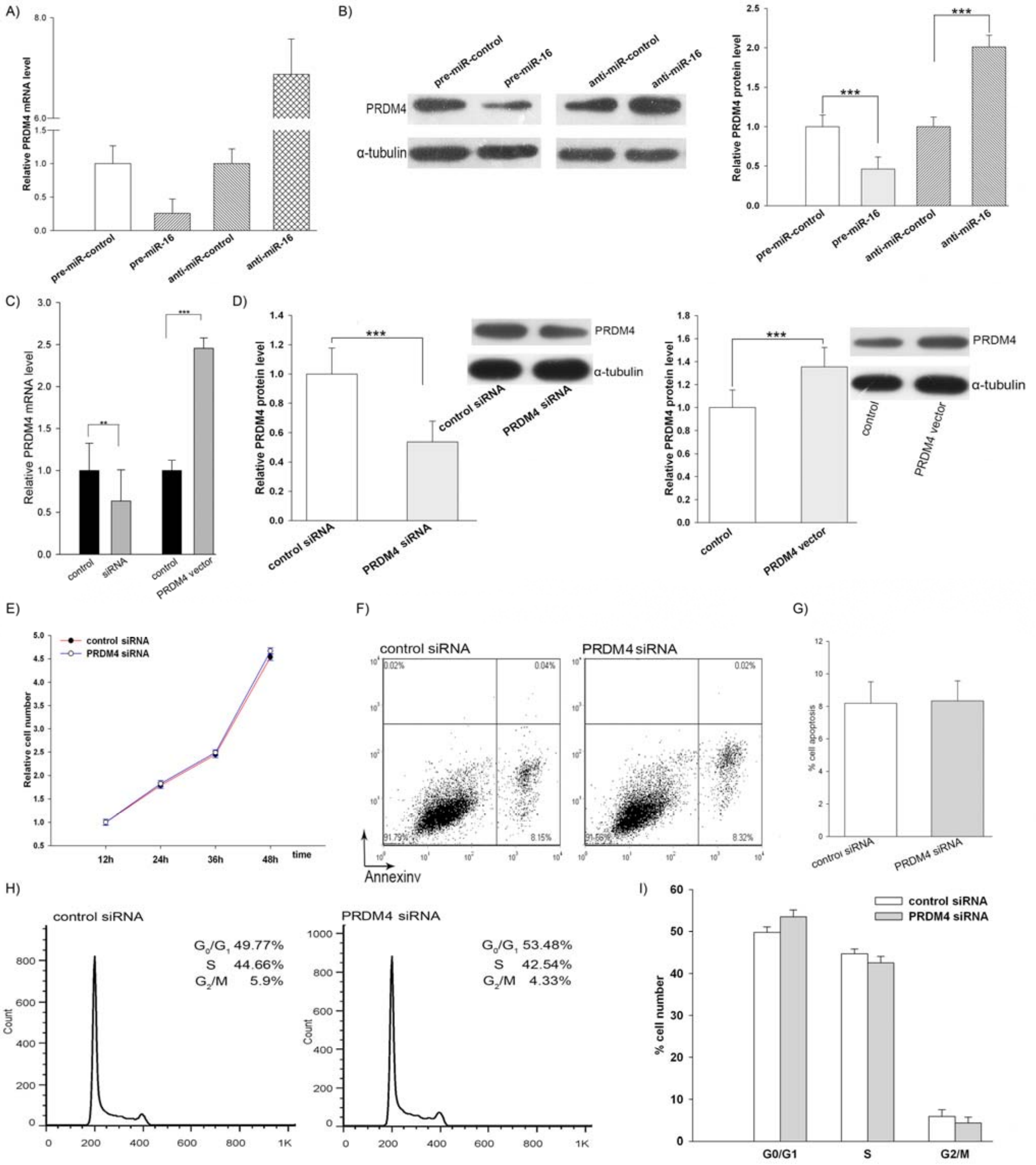
A)
Relative PRDM4 mRNA level
pre-miR-control
pre-miR-16
anti-miR-control
anti-miR-16
B)
PRDM4
α-tubulin
Relative PRDM4 protein level
C)
Relative PRDM4 mRNA level
control
siRNA
PRDM4 vector
D)
control siRNA
PRDM4 siRNA
control
E)
control siRNA
PRDM4 siRNA
Relative cell number
12h
24h
36h
48h
time
F)
Annexinv
G)
% cell apoptosis
H)
Count
G0/G1 49.77%
S 44.66%
G2/M 5.9%
G0/G1 53.48%
S 42.54%
G2/M 4.33%
I)
% cell number
G0/G1
S
G2/M

Figure S1

Table S1. mRNAs identified as downregulated in cells overexpressing miR-16 by this study and our previous study[1].

	The present study					The previous study		
Gene name	A549	MCF-7	HEK-293	Hela	SW480	A549	MCF7	HEK-293
ARL2	√	√	√		√	√	√	√
CDS2	√	√	√	√		√	√	√
ANAPC13		√	√	√	√	√	√	√
MAP7	√	√	√	√	√	√	√	√
ARG2	√	√	√		√	√	√	√
RTN4	√	√	√		√	√	√	√
RARS	√	√	√	√		√	√	√
SPRYD3	√	√	√	√	√	√	√	√
RPS6KA3	√	√	√	√	√	√	√	√
NUPL1		√	√	√	√		√	√
PRDM4	√	√	√	√	√	√		
ATF6	√	√	√		√	√		√
TMEM109		√	√	√	√			√
BCR		√	√	√	√			√
KIF3B	√	√	√	√	√	√		√
ENTPD6	√	√	√	√	√	√	√	
CCND3		√	√	√	√		√	√
ARHGDIA		√	√	√	√			√
GABARAPL1		√	√	√	√			√
PLEKHB2		√	√	√	√		√	√
FLJ11149		√	√	√	√		√	√
DNAJC5		√	√	√	√	√		
ZNF622		√	√	√	√	√		√
ANXA11	√	√	√		√	√		
VPS33B	√	√	√		√	√		√
ANLN		√	√	√	√		√	
PTH2	√	√	√		√	√		
SLC35A4	√	√	√			√	√	√
C1orf2		√	√		√	√	√	√
VTI1B	√	√	√			√	√	√
RNF111	√	√	√			√	√	√
ALG3	√	√	√			√	√	√
XKR8		√	√			√	√	√
MRPL20	√	√	√			√	√	√
MMS19	√	√	√			√	√	√
CCNE1		√	√		√	√	√	√
TOMM34		√	√			√	√	√

CHPT1		√	√	√	√	√	√
KIAA0746	√	√		√	√	√	√
CXorf40A	√	√	√		√	√	√
NECAP1	√	√	√		√	√	√
PPP1R11		√	√		√	√	√
KIF1B	√	√		√	√	√	√
SMURF2		√	√		√	√	√

The mark "√" means that the mRNAs showed downregulation (fold change < 0.66) in response to miR-16 overexpression. The 9 mRNAs selected by both this study and our previous study were marked in red. The 18 mRNAs selected only by this study were marked in green, and 17 mRNAs selected only by our previous study were marked in blue.

[1]Wang K, Li P, Dong Y, Cai X, Hou D, Guo J, Yin Y, Zhang Y, Li J, Liang H, Yu B, Chen J, Zen K, Zhang J, Zhang CY, Chen X: **A microarray-based approach identifies ADP ribosylation factor-like protein 2 as a target of microRNA-16.** *J Biol Chem* 2011, **286**:9468-9476.
